# A pilot study examining the use of Goal Management Training in individuals with obsessive-compulsive disorder

**DOI:** 10.1186/s40814-020-00684-0

**Published:** 2020-10-06

**Authors:** Duncan H. Cameron, Randi E. McCabe, Karen Rowa, Charlene O’Connor, Margaret C. McKinnon

**Affiliations:** 1 Anxiety Treatment and Research Clinic, St. Joseph’s Healthcare Hamilton, Hamilton, ON Canada; 2 Department of Psychiatry and Behavioural Neurosciences, McMaster University, Hamilton, ON Canada; 3 Homewood Research Institute, Guelph, ON Canada; 4 Mood Disorders Program, St. Joseph’s Healthcare Hamilton, Hamilton, ON L8P 3R2 Canada

## Abstract

**Background:**

Recent meta-analyses point towards cognitive impairments in obsessive-compulsive disorder (OCD), particularly in such executive function subdomains as planning and organization. Scant attention has focused on cognitive remediation strategies that may reduce cognitive dysfunction, with a possible corresponding decrease in symptoms of OCD.

**Objective:**

The aim of this study was to assess the implementation of a standardized cognitive remediation program, Goal Management Training (GMT), in a pilot sample of individuals with OCD.

**Method:**

Nineteen individuals with a primary DSM-5 diagnosis of OCD were randomized to receive either the 9-week GMT program (active group; *n* = 10) or to complete a 9-week waiting period (waitlist control; *n* = 9). Groups were assessed at baseline, post-treatment, and 3-month follow-up. The assessment comprised neuropsychological tasks assessing a variety of cognitive domains, and subjective measures of functioning and of symptom severity.

**Results:**

The active condition showed significant improvements from baseline to post-treatment on measures of inattention, impulsivity, problem-solving, and organization compared to controls. Moreover, whereas the active group reported a significant improvement in subjective cognition over the course of treatment, no such improvement emerged in the waitlist group over this same period. Neither group showed improvement on indices of depressive, anxiety, or OCD-related symptom severity.

**Discussion:**

The results of this small pilot investigation indicate that, although promising, this protocol requires several modifications to be best suited for this population. Replication of these findings is awaited, with current results potentially limited by sample characteristics including motivation to seek and complete treatment, and high attrition at 3-month follow-up (*n* = 6 completers).

**Trial Registration:**

NCT02502604. (December 7, 2018)

## Introduction

Obsessive-compulsive disorder (OCD) is characterized by the presence of recurrent obsessions and/or compulsions that cause marked anxiety and interfere with daily functioning [1]. OCD affects between 2 and 3% of adults and about 1–2% of adolescents and children [1–3], making it approximately twice as prevalent as schizophrenia or bipolar disorder [4]. The neuropsychology of OCD has received considerable attention, with more than 250 peer-reviewed articles published in the last quarter century exploring cognitive performance in this disorder [5]. Individuals with OCD show poor performance across multiple cognitive domains, including on measures of memory and of executive function, with these impairments likely being involved in the etiology and maintenance of symptoms [6–12].

In most cases, untreated OCD runs a chronic and deteriorating course. Of individuals diagnosed with OCD, 84% have a chronic course and 14% experience a deteriorating illness [13]. In light of its prevalence and associated personal and societal costs, OCD is a significant public health concern in Canada, rendering identification and development of effective, evidence-based treatment approaches critically important. Despite well-accepted treatment options such as SSRIs and cognitive-behavioral therapy [14], a successful outcome in OCD is defined typically as a reduction in symptom severity of 25–50% [15]; the majority of “responders” to first-line treatments are left with residual symptoms that are clinically relevant and disabling.

OCD has been linked neurobiologically to altered functioning in cortico-striato-thalamo-cortical (CSTC) circuits, with the fronto-striatal network thought strongly implicated in the poor performance observed, relative to healthy controls, on measures of executive functioning in this disorder [16]. Consistent with alterations in these circuits, recent meta-analyses reveal primarily medium effect sizes on measures of response inhibition (Cohen’s *d* = − .24 to − .49), planning (*d* = − .44), set shifting (*d* = − .32 to − .52), and processing speed (*d* = − .34 to − .52) [17, 18] in OCD. Finally, several studies point towards poor performance on measures of cognitive flexibility/planning and motor inhibition relative to matched controls among unaffected first-degree relatives [19–21], suggesting that alterations in executive functioning might represent a cognitive endophenotype of this illness that is consistent with its proposed neurobiological basis.

Interestingly, despite knowledge of reduced cognitive performance in OCD and its potential to exert deleterious effects on treatment and on functional outcomes, to date, cognitive remediation has received scant attention in the OCD literature. Here, two studies reported positive effects of cognitive retraining strategies for organizational impairment. In one, OCD participants were trained briefly on organizational strategies, and when assessed using the Rey-Osterrieth Complex Figure Task (RCFT), the training group showed significantly greater organization and accuracy scores compared to a non-training group [22]. In a second study, Park et al. [23] used a revised version of the Wechsler Adult Intelligence Scale (WAIS) block design task as a training tool and aided participants in applying problem-solving and organizational strategies to everyday life over the course of nine 60-min sessions. The authors found that memory function in the treatment group improved and that clinical symptoms were reduced after training when compared to a matched control group. Although neither of these studies employed an established protocol, they point to the potential of cognitive remediation, focusing on improvement of organizational and planning strategies, as a treatment strategy for patients with OCD. Notably, despite an emphasis on applying organizational and problem-solving strategies, both interventions described here targeted focused skills with the primary aim of improving performance on specific neuropsychological tasks (organizational strategy on either RCFT or WASI block design). Although effective for improving task-specific performance, these approaches often lack the ability to show generalizable improvements.

Goal Management Training (GMT) is a staged cognitive remediation program aimed at recovery of executive function and goal-directed behavior [24]. This program is unique in applying a “top-down” approach, focusing primarily on higher-order executive function domains and teaching skills (such as goal-setting and monitoring progress) aimed at regulating these systems. The program is traditionally offered in a group setting, with the expectation of similar group-related benefits as are seen in group CBT [25], such as accountability, normalization of experience, and sharing of experiences. Here, participants are expected to leave treatment with strategies that can be applied to a variety of daily tasks, ideally improving not only performance on neuropsychological tasks but also leading to improvements in daily functioning, which can offer significant benefits over the task-specific approaches mentioned above. The efficacy of this approach, both as a stand-alone treatment and a supplementary treatment to psychotherapy, has been demonstrated in clinical and non-clinical populations that experience deficits in executive functioning, attention, and memory. A recent meta-analysis of GMT covering 21 studies with 19 separate treatment populations found significant small-to-moderate effects across a wide range of executive function, working memory, and long-term memory tasks, further suggesting that GMT offers an effective cognitive remediation intervention [26]. Critically, when assessed at follow-up, the effects of GMT on executive function task performance were maintained (Hedges’ *g* = .549). Subjective reports of executive function, however, were not a finding that requires further investigation.

GMT targets primarily the brain’s sustained attention system, regulated by several regions including dorsolateral prefrontal cortex, posterior parietal and thalamic regions [27–29] collectively implicated in executive functioning, and higher-order attentional processing. The cognitive strategies trained in GMT are designed to facilitate the resumption of executive control and a reinstatement of self-regulatory goals. Critically, the current CSTC model of the neurobiology of OCD points towards poor performance and related slowness on measures of executive function assessing response inhibition, decision making, task switching, and planning in association with dysregulation in related regions including dorsolateral prefrontal cortex and orbitofrontal cortex with these subdomains serving in concert to regulate complex behavior. Accordingly, we predicted that GMT, targeting selectively neural regions and associated cognitive functions implicated in the CTSC model of OCD, and with demonstrated success in remediating poor performance in clinical populations with executive dysfunction, would be effective in reducing cognitive performance deficits in OCD and in improving daily functioning.

Hence, the primary aim of the present study was to conduct a novel pilot investigation of the application of a cognitive remediation program aimed at improving goal-directed behaviors that are dependent executive functioning in individuals with DSM-5-diagnosed OCD. Specifically, we aimed to examine the suitability of a well-established, 9-week group cognitive training program, Goal Management Training, and to assess whether this protocol results in a significant improvement in performance in the cognitive domains of executive function, attention, and memory in an OCD sample. An additional objective was to collect participant feedback about the treatment protocol to evaluate which changes should be considered should the protocol be adapted for a full-scale clinical trial in future. We hypothesized that the GMT program would be well-received and would lead to significant improvements in performance on tasks related to planning, organization, and attention from pre- to post-treatment relative to no such improvements in waitlist controls over the same time period. Further, we expected that the GMT group will show significant improvement on ratings of subjective cognition and of functional outcomes, relative to waitlist controls.

## Method

### Participants

Nineteen (*n =* 19) participants with a principal diagnosis of DSM-5 OCD were recruited from the Anxiety Treatment and Research Clinic at St. Joseph’s Healthcare Hamilton. Inclusion criteria included: (1) between the ages of 18 and 60 years; (2) experiencing clinically significant obsessive-compulsive symptoms based on the Yale-Brown Obsessive Compulsive Scale (Y-BOCS), score > 17; (3) if on medications for OCD, on stable dose for a minimum of 8 weeks prior to initiation of the study; (4) must not have completed > 8 sessions of CBT for OCD in the last 6 months, and must refrain from participation in CBT throughout the duration of the study; and (5) are able to provide written informed consent. Exclusion criteria include: (1) a concurrent diagnosis of a severe mood disorder, schizophrenia, or other psychotic disorders, or substance abuse/dependence; (2) suspected organic pathology; (3) active comorbid medical condition that might require urgent intervention during the course of treatment; and (4) a history of traumatic brain injury or concussion/loss of consciousness.

### Experimental design and procedure

Participants were assigned randomly (using https://www.graphpad.com/quickcalcs/randomize1/) to receive: (1) a 9-week structured cognitive remediation program, GMT; or (2) a 9-week waitlist condition. Participants were assessed at baseline, post-treatment, and 3-month follow-up. The experimental design is a 2 (treatment condition) by 3 (assessment phase) repeated-measures factorial design. Participants randomized to the waitlist condition were informed that they would have the opportunity for therapist-led GMT group treatment at the end of the study.

Participants were introduced to the study by research staff at the point of referral to our clinic, and those interested in hearing a detailed explanation were invited to speak with a research assistant to review the study in detail and obtain informed consent. Baseline assessments were completed within 14 days of initial contact. At study entry, participants completed a battery of symptom and subjective cognition measures. Participants also completed neuropsychological testing to assess executive functioning, attention, and memory (see below), as well as several functional outcome measures. Trained researchers at the graduate level or higher administered the neuropsychological testing and were blind to research condition. All measures were completed/administered at baseline, post-treatment, and 3-month follow-up.

### Study conditions

#### Goal Management Training

GMT is a structured, short-term, present-oriented cognitive remediation program with an emphasis on mindfulness and practice in planning and goal-oriented behaviors. The primary objective of GMT is to train patients to stop ongoing behavior in favor of executive control in order to define goal hierarchies and monitor performance. This is achieved through nine weekly 2-h sessions, including instructional material, interactive tasks, discussion of patients’ real-life deficits, and homework assignments. Each of the nine GMT sessions is detailed further in Table 1. Mindfulness meditation is also incorporated for the purpose of developing the skill of bringing one’s mind to the present to monitor ongoing behavior, goal states, and the correspondence between them. The program also incorporates real-life examples provided by the group facilitator and the participants to illustrate goal attainment failures and successes, as well as in-session practice on complex tasks that mimic real-life tasks that are problematic for individuals with executive function deficits (such as planning and set-shifting tasks).

**Table 1:** Overview of Goal Management Training protocol

GMT session	Description
Session 1: the absent mind, the present mind	Introduce the concept of absentmindedness and normalize the experience. Explain present-mindedness using mindfulness techniques.
Session 2: absentminded slip-ups	Introduce construct of absentminded slips with examples, and discuss emotional and practical consequences. Introduce the “Body Scan” mindfulness exercise.
Session 3: the automatic pilot	Describe “automatic pilot” as being a habitual mechanism which can lead to inappropriate responses or actions if not monitored. Introduce the “Breathing Exercise” mindfulness technique.
Session 4: stop the automatic pilot	Participants are introduced to the “STOP!” technique as a method of bringing one’s attention to the present to monitor current behavior. The short “Breath Focus” mindfulness exercise is described.
Session 5: the mental blackboard	The construct of working memory as a “mental blackboard,” which can be erased or over saturated with information, is explained. Participants are taught to check “the mental blackboard” to keep current goals at the forefront of memory. Introduce how to incorporate present-mindedness (specifically the “Breath Focus”) into behavior monitoring and executing difficult tasks as a method for increasing accuracy and memory.
Session 6: state your goal	Describe how goals can become entangled when attempting to multi-task. Introduce the concept of stating one’s goal as a way to aid encoding and recall of that goal.
Session 7: making decisions	Introduce the concept of conflicting goals and detail strategies for how to make decisions. Review methods for keeping track of complex goals using to-do lists.
Session 8: splitting tasks into subtasks	Practice completing tasks that are too complex to rely on working memory only, and detail strategies for how to divide large goals into a series of smaller, more manageable subgoals.
Session 9: STOP!	Review the material covered across previous sessions and underscore the importance of goal monitoring (the “STOP!” technique).

In the present study, participants who terminated treatment before completing 55% (or 5/9) of the GMT sessions were considered “drop-outs.” This value was determined by expert opinion of clinicians experienced with the GMT protocol. Based both on the repetitive nature of the session content and the modest cognitive deficits expected in this population, it was deemed necessary for completers to have attended the majority of the sessions, rather than the entirety. In the present study, one participant was counted as a drop-out as defined by these criteria, and one further patient was required to drop-out due to admission to an inpatient program; data were not included in the analysis for either of these cases. A total of *n =* 12 participants were randomized to the active condition, and a total of *n* = 10 completed treatment and the post-treatment assessment. The mean number of sessions attended by this group was 7.2 (SD = 1.4).

### Waitlist control group

Individuals randomized to this group (*n* = 9) were required to wait 9 weeks without participating in traditional CBT (or other psychotherapy) for OCD, and, if on medication, must remain on stable dosage for the duration of the study. Upon completion of the follow-up assessment, participants were invited to commence GMT at our clinic.

### Measures and materials

#### Symptom measures

Yale-Brown Obsessive Compulsive Scale (Y-BOCS) [30, 31].The Y-BOCS is a standardized rating scale measuring 10 items pertaining to obsessions and compulsions on a 5-point Likert scale ranging from 0 (no symptoms) to 4 (severe symptoms). Both the self-report and clinician interview versions of the Y-BOCS have been shown to possess high internal consistency and validity.

Depression, Anxiety and Stress Scales (DASS-21) [32]. The DASS-21 is a set of three self-report scales designed to measure depression, anxiety, and stress. Each subscale consists of seven items rated on a scale from 0 (did not apply at all) to 3 (applied to me very much).

#### Subjective cognition

Participants completed three, brief self-report measures addressing cognitive performance. The *Cognitive Failures Questionnaire (CFQ )* [33] captures daily errors in distractibility, blunders, names, and memory. The *Dysexecutive Questionnaire (DEX )* [34] involves self- and informant-ratings of inhibition, positive and negative affect, memory, and intention. The *memory and cognitive confidence scale (MACCS )* [35] involves several questions about confidence in one’s own memory and was developed for use in OCD.

### Functional outcome measures

Participants completed several self-report measures assessing functional outcomes. The *WHO Disability Assessment Scale (WHODAS) 2.0* is a 36-item questionnaire which assesses an individual’s ratings of their own performance across domains of cognition, mobility, self-care, getting along, life activities, and participation in social activities [36]. The *Illness Intrusiveness Rating Scale* (IIRS [37];) is a 13-item instrument providing ratings of quality of life over three domains including relationships and personal development, intimacy, and instrumentals. Finally, the *Sheehan Disability Scale (SDS)* is a brief measure of disability in work, social relationships, and family life [38].

### Neuropsychological assessment

Here, we assessed several, separable cognitive domains that are sensitive to OCD. Attention and response inhibition were measured using *Conners’ Continuous Performance Task* (CPT) [39]. The *Stroop Color and Word Test* [40] assesses processing speed (color and word reading) and sensitivity to suppress habitual responses (interference trial). The *Tower of London* [41] task requires participants to match a pattern on a board with three pegs of different sizes, and involves aspects of planning, organization, and problem-solving. Verbal memory was assessed using the *California Verbal Learning Test—Second Edition* (CVLT-II) [42], which provides indices of immediate and delayed memory performance, interference learning, and recognition. The *Wechsler Test of Adult Reading* [43] was used to estimate pre-morbid intellectual functioning.

### Qualitative interview

A qualitative exit interview designed for this study was implemented to gather participant opinions of the GMT program and protocol. This interview was conducted at post-treatment and consisted of a mixture of 11 open- and closed-ended questions concerning opinions about various aspects of the program and how helpful participants found it.

### Data analysis

Only six of nineteen (32%) of participants completed the 3-month follow-up assessment, with the primary reason for low rate of completion being refusal to return for the final assessment visit. Accordingly, the primary outcome analyses for this study were the analyses measuring change from pre- to post-treatment. Repeated measures ANOVAs were used for all outcome variables with estimates of partial eta-squared for effect size (interpreted conservatively as small = .01, medium = .10, and large = .25). Simple main effects are reported for those results where an interaction was significant. To avoid further risk of type I error due to multiple comparisons, 2 × 3 ANOVAs for follow-up data were only completed for significant pre/post-interactions or main effects of group. Results from the qualitative exit interview were summarized using percentages. Reponses to open-ended questions were evaluated for similar themes, which were then grouped together and represented as percentages. All analyses were conducted using IBM SPSS version 23.

## Results

The mean age of participants was 45.5 (SD = 12.6), and 55% were female. The average level of education was 14.6 years (SD = 2.3) indicating completion of at least some college or university for much of the sample. The mean duration of OCD since onset of symptoms was 23.7 years (SD = 12.4) which is reflective of the mean age and generally early onset of OCD typical of this disorder. The mean Y-BOCS total score was 21.2 (SD = 5.6) which is expected given that 74% of the sample were using SSRIs and/or had recently completed a course of CBT for OCD. There were no significant differences between the waitlist control and active group participants at baseline on any demographic variables. The mean WTAR raw score was 41.6 (SD = 6.7), and the mean WTAR standard score was 113.2 (SD = 10.4) indicating slightly above average estimates of pre-morbid intelligence at baseline for the combined active treatment group and waitlist group.

### Neuropsychological assessment

Means and standard deviations for significant results are presented in Table 2; a full list of all variables tested is presented in Table 3.

**Table 2:** Means and standard deviations (SDs) for statistically significant 2 × 2 repeated measures ANOVAs for GMT (*N* = 10) versus WLC (*N* = 9)

		Pre-treatment		Post-treatment	
Variable	Group	Mean	SD	Mean	SD
Neuropsychological assessment					
Stroop Color-Word T score (MEs of time and group)	GMT	42.9	6.1	48.3	7.1
	WLC	50.0	8.3	51.8	8.1
TOL total correct SS (group X time and ME of time)	GMT	100.0	14.1	114.2	16.8
	WLC	93.1	15.8	98.4	15.8
TOL initiation time SS (group X time)	GMT	108.8	18.4	121.2	18.6
	WLC	105.1	12.5	104.1	8.8
CPT commission errors T score (ME of time)	GMT	54.1	9.2	50.2	8.5
	WLC	52.2	7.8	48.6	8.1
CPT hit reaction time T score (group X time)	GMT	60.2	11.2	53.2	7.2
	WLC	52.3	7.4	52.5	8.1
Functional outcomes					
SDS work (group X time)	GMT	3.8	2.9	3.1	2.9
	WLC	3.0	1.9	3.3	2.1
SDS social (group X time)	GMT	5.1	3.4	4.5	3.5
	WLC	2.9	1.7	4.0	2.1
SDS family (group X time)	GMT	4.8	2.5	4.2	2.4
	WLC	3.2	2.4	4.0	2.2
IIRS instrumental subscale (group X time)	GMT	15.3	5.3	13.6	4.8
	WLC	11.4	2.9	12.6	2.9
IIRS Total Score (group X time)	GMT	49.1	15.1	43.3	11.6
	WLC	35.2	10.3	37.4	9.4
WHODAS 2.0 understanding (group X time and ME of time)	GMT	6.7	4.0	5.4	4.0
	WLC	6.5	2.9	6.6	3.2
Subjective cognition					
MACCS Total Score (MEs of time and group)	GMT	92.9	9.6	84.1	13.7
	WLC	76.8	7.7	74.6	10.3
MACCS general memory (MEs of time and group)	GMT	44.2	8.7	38.9	8.0
	WLC	33.4	6.8	29.8	7.6
CFQ (group X time and ME of time)	GMT	45.1	10.4	35.3	9.2
	WLC	43.4	8.1	44.3	7.9

The value in parentheses below each variable is the result provided by 2 × 2 repeated measures ANOVA.

*CFQ* Cognitive Failures Questionnaire, *CPT* Conners’ Continuous Performance Task, *GMT* Goal Management Training group, *IIRS* Illness Intrusiveness Rating Scale, *MACCS* Memory and Cognitive Confidence Scale, *ME* main effect, *TOL* Tower of London, *WHODAS* World Health Organization Disability Assessment Schedule, *WLC* waitlist control group

**Table 3:** Complete list of assessment measures

Measure	
Neuropsychological assessment	
California Verbal Learning Test–Second Edition	
Stroop Task	
Tower of London	
Conners’ Continuous Performance Task – Second Edition	
Wechsler Test of Adult Reading (baseline only)	
Functional outcome measures	
Sheehan Disability Scale	
Illness Intrusiveness Rating Scale	
WHO Disability Assessment Schedule 2.0 (36-item self-report)	
Subjective cognition measures	
Memory and Cognitive Confidence Scale	
Cognitive Failures Questionnaire	
Dysexecutive Questionnaire	
Symptom measures	
Yale-Brown Obsessive-Compulsive Scale	
Depression Anxiety and Stress Scales 21-item	

### Planning and problem-solving

There was a significant group X time interaction for the total number of problems solved correctly on the Tower of London (*F*(1,17) = 4.6, *p =* .047) as well as a main effect of time (*F*(1,17) = 20.7, *p* < .001). Simple main effect analysis revealed that whereas the GMT group showed a significant improvement from baseline to post-treatment (*F*(1,17) = 22.6, *p* < .001, *η*^2^_p_ = .571), the waitlist group did not (*F*(1,17) = 2.8, *p* = .108, *η*^2^_p_ = .145). There was also a group X time interaction for TOL initiation time (*F*(1,17) = 18.4, *p* < .001) with simple main effects revealing an increase in initiation time from baseline to post-treatment in the treatment group (*F*(1,17) = 8.1, *p =* .011, *η*^2^_p_ = .324) but not in the waitlist controls (*F*(1,17) = .48, *p =* .83, *η*^2^_p_ = .003).

### Attention and processing speed

The number of commission errors on the CPT was lower at post-treatment compared to baseline for both groups, represented by a marginally significant main effect of time (*F*(1,16) = 3.9, *p =* .06, *η*^2^_p_ = .199). There was also a significant group X time interaction for Hit Reaction Time (*F(1,16)* = 47.1, *p =* .017). Simple main effects revealed that there was a significant reduction in reaction time to correct responses for the GMT group (*F*(1,16) = 4.8, *p =* .043, *η*^2^_p_ = .232) but not for those in the waitlist control group (*F*(1,16) = .003, *p =* .96, *η*^2^_p_ = .000). No additional differences emerged on the CPT.

### Verbal memory

There was a main effect of time for number of correct responses on CVLT Trial 1 (*F*(1,17) = 9.2, *p =* .007, *η*^2^_p_ = .351) and long delay free recall (*F*(1,17) = 10.2, *p =* .005, *η*^2^_p_ = .375), indicating improved performance for both groups from baseline to post-treatment. No other significant differences emerged on the CVLT.

### Cognitive interference

The Stroop Task did not yield any significant differences at a critical level of *α* = .05. There were, however, trends toward a main effect of group (*F*(1,17) = 3.3, *p =* .085, *η*^2^_p_ = .165) and of time (*F*(1,17) = 3.3, *p =* .084, *η*^2^_p_ = .165) for the Color-Word trial, with both groups improving over time and the waitlist group having slightly higher scores at both time points (see Table 2).

### Functional outcomes

All functional measures are scored such that decreases in scores indicate improved functioning. There was a trend toward group X time interactions on the work (*F*(1,17) = 3.6, *p =* .076, *η*^2^_p_ = .173), social (*F*(1,17) = 3.8, *p =* .066, *η*^2^_p_ = .185), and family (*F*(1,17) = 3.6, *p =* .073, *η*^2^_p_ = .176) subscales of the Sheehan Disability Scale. Simple main effects were not calculated for these marginally significant results; however, means and SDs displayed in Table 2 illustrate that whereas participants’ scores on these disability subscales generally trended downward from baseline to post-treatment in the GMT group they tended to increase over time in the waitlist control group.

There were significant group X time interactions for the instrumental subscale (*F*(1,17) = 3.6, *p =* .076, *η*^2^_p_ = .173) and total score (*F*(1,17) = 3.6, *p =* .076, *η*^2^_p_ = .173) of the IIRS. Here, simple main effects revealed that whereas the scores of individuals in the GMT group improved significantly from baseline to post-treatment on the IIRS instrumental subscale (*F*(1,17) = 3.9, *p =* .046, *η*^2^_p_ = .188), no such improvement was observed in the waitlist control group (*F*(1,17) = 1.5, *p =* .236, *η*^2^_p_ = .082). This pattern was also observed for the IIRS total score (GMT: *F*(1,17) = 6.8, *p =* .018, *η*^2^_p_ = .286; waitlist: *F*(1,17) = .727, *p =* .406, η^2^_*p*=_.041). The WHODAS 2.0 total score measuring subjective ratings of overall functioning in daily activities decreased significantly for both groups (main effect of time (*F*(1,17) = 5.2, *p =* .036, *η*^2^_p_ = .234). There was also a significant main effect of time (*F*(1,17) = 4.8, *p =* .042, *η*^2^_p_ = .222) and a significant group X time interaction (*F*(1,17) = 4.8, *p =* .042, *η*^2^_p_ = .222) for the understanding subscale, which contains items related to cognition and understanding while communicating with others. Whereas simple main effects revealed a significant decrease in scores over time for the GMT group (*F*(1,17) = 10.2, *p* = .005, *η*^2^_p_ = .376), no such improvement emerged in the waitlist group (*F*(1,17) = 0, *p* = 1.0, *η*^2^_p_ = .000).

### Subjective cognition measures

There were significant main effects of group and time for the MACCS total score (group: *F*(1,17) = 8.2, *p =* .011, *η*^2^_p_ = .222; time: *F*(1,17) = 7.9, *p =* .012, *η*^2^_p_ = .222) and the general memory subscale (group: *F*(1,17) = 8.9, *p =* .008, *η*^2^_p_ = .222; time: *F*(1,17) = 11.7, *p =* .003, *η*^2^_p_ = .222), revealing higher scores in the GMT group, and improved scores in both groups over time.

There was also a significant group X time interaction (*F*(1,17) = 9.2, *p =* .007) and main effect of time (*F*(1,17) = 6.4, *p =* .022) for the CFQ total score. Simple main effects revealed that overall ratings of subjective cognition improved significantly over the course of treatment for the GMT group (*F*(1,17) = 16.3, *p* = .001, *η*^2^_p_ = .490) but not the waitlist control group (*F*(1,17) = .121, *p* = .732, *η*^2^_p_ = .007). There were no significant differences on the DEX.

### Symptom severity

There were no differences observed between time points or groups on either the Y-BOCS or the DASS-21 (total and subscale scores).

### Three-month follow-up data

Follow-up data were only available for *n* = 6 participants (three waitlist and three GMT). No additional differences were seen when a set of 2 × 3 ANOVAs were run for these participants. When carrying forward the significant results from the 2 × 2 model to the 2 × 3, all significant main effects became insignificant.

### Qualitative exit interview

Of the (*n* = 10) participants who completed the GMT program, *n* = 9 completed the qualitative interview. Of these participants, two (22%) stated that the program was helpful for reducing OCD symptoms, while five (56%) responded that the program helped them feel better in day-to-day activities. When asked which aspect of the program they found most helpful, seven (78%) reported the STOP! technique (learning to take time to think before acting), and all (100%) participants reported that the mindfulness techniques were the least helpful. Providing feedback on the program, eight individuals (89%) stated that the content was too simple or slow-moving. Participants also reported that the homework assignments were helpful for applying the techniques to real-life situations (78%) but 67% responded that they did not fully commit to practicing the assignments at home. When asked what could be done, if anything, to make the program more effective, 78% stated that the material could be condensed into fewer sessions, and 67% requested that there be more OCD-specific content. All of the participants reported that they felt they would be able to apply the skills from this program to their everyday lives. Seven of nine participants stated that they planned to continue using the GMT skills in future (the other two said “probably”) while only 33% reported plans to continue mindfulness practice. Three participants stated that they would recommend this program to others, while five of the remaining participants said they would recommend the program if someone was experiencing difficulty with memory or concentration.

## Discussion

The results from this study indicate that GMT has the potential to serve as a cognitive remediation program for individuals with OCD, but that several revisions might be required to adapt this program to best serve the needs of this population. Analyses showed focused, significant improvements in cognitive functioning for individuals in the GMT program relative to those in the waitlist control group. Specifically, improvements in performance on neuropsychological tasks assessing planning and impulsivity (TOL initiation time), problem-solving (TOL total problems solved correctly), and inattention and processing speed (CPT hit reaction time) were observed for the GMT group from pre- to post-treatment but not for those in the waitlist control group. Effect sizes for significant simple main effects ranged from .188 (medium) to .571 (large) indicating promise for these variables in a large-scale trial. Even for non-significant results, which can be expected given the under-powered nature of a pilot investigation, 64% of variables achieved at least a small effect (*η*^2^_p_ > .01), 25% of which were medium effects (*η*^2^_p_ > .10). Based on an average effect size of *η*^2^_p_ = .054 for neuropsychological variables and *η*^2^_p_ = .161 for functional outcomes observed across all results in this study (significant and non-significant), with a critical alpha = .05 and 80% power, a sample size of *n* = 23 per group (for functional measures) and *N* = 23 (for neuropsychological variables) would be required for future full-scale trials to reliably detect the smallest desired effects.

While they should be interpreted with caution given the pilot sample, these results are in keeping with the emphasis placed by GMT on planning, problem-solving, and attention, executive functioning subdomains putatively affected in this population. Subjective report indicated that participants in the GMT group reported a decrease in how severely their OCD symptoms affected their lives both overall (IIRS total score) and on items related to daily functioning (IIRS Instrumental subscale), as well as improved outcomes related to daily cognitive functioning (WHODAS Understanding subscale) and general day-to-day tasks (WHODAS total score).

Critically, given that no significant differences were observed for any symptom measures in the active treatment and waitlist group, it seems likely that the functional improvements seen here are attributable primarily to the cognitive remediation program itself. Notably, there were marginally significant improvements from pre- to post-treatment for GMT relative to waitlist for each of the work, social, and family subscales of the Sheehan Disability Scale, suggesting reductions in subjective disability across these three areas. Finally, the GMT group only showed significant improvement in subjective cognition as rated by the Cognitive Failures Questionnaire. Although we failed to find that positive effects of GMT were maintained at follow-up, it is probable that this negative finding stemmed from the small number of participants (*N* = 6) available for follow-up testing.

Taken together, the findings reported here support the “top-down” nature of the Goal Management Training protocol in that those variables showing significant effects due to treatment were related largely to executive control systems. Moreover, the performance gains observed hold the potential to be clinically meaningful given the typical presentation of cognitive deficits in the areas of response inhibition, decision-making, task switching, and planning observed in individuals with OCD, as proposed by the CSTC model [44]. Here, given the potential association between cognitive dysfunction and reduced treatment response to pharmacological and non-pharmacological interventions in OCD [45–48], it is possible that cognitive interventions such as GMT may be best positioned prior to the onset of standard behavioral approaches such as CBT, thus enhancing the potential to benefit from these standard approaches.

Results of the qualitative exit interview revealed that although participants seemed generally to enjoy the program and noticed subjective benefits, they also felt that the program was too long and the material was slow moving. Here, cognitive deficit observed in individuals with OCD—despite medium-to-large effects reported for some domains in meta-analyses [17, 18]—tend to be subtle. Thus, gains attributable to GMT are likely to be perceived as equally subtle by participants, thus increasing the likelihood that patients take more notice of, for example, the repetition of concepts. Given that GMT remains one of few established cognitive remediation protocols available, addressing the pacing and content layout of the program to be more specific to this particular population would make it an excellent candidate for future therapeutic and research applications in OCD. In order to be best suited to this population, a shorter format of 4–5 sessions, with more content covered per session and less repetition of previous concepts is perhaps more appropriate.

In the present study, results of pre- and post-test measurements of neuropsychological functioning are reported using normative values. Inspection of the mean standard scores or *T* scores in Table 2 reveals that for those neuropsychological domains showing improvement, participant scores tended to improve from the average to high-average range of performance. Here, the GMT group showed an improvement from the average to high average performance for the total number of problems solved correctly on the Tower of London that was accompanied by a corresponding increase in problem initiation time (high average to superior performance) reflecting increased inhibitory control. Scores on the Stroop task color-word trial were also seen to increase from low average to average in the GMT group. Similarly, for sustained attention, for the CPT mean hit reaction time, the GMT group’s scores changed from slow to average compared to no change for the waitlist group.

Interestingly, a recent study by Moritz et al. [49] suggests that poor neuropsychological performance in OCD may be mediated, in part, by obsessive-compulsive symptoms—for example, an individual with symmetry obsessions might take longer to perform a task not because of neuropsychological impairment but because of the need to achieve symmetry with the items used in that task and a decrease in motivation, two domains not measured in this study. Notably however, performance time *increased* on a measure of inhibitory control (Tower of London initiation time) in the GMT group only, suggesting that a simple increase in reaction time on neuropsychological measures cannot account solely for poor pre-intervention performance in this OCD sample.

Further inspection of Table 2 reveals that although the GMT and wait list groups did not differ statistically on any objective measures at baseline, nor were there any significant main effects of group only (though attention should be drawn to the main effects of group and time for the MACCS), participants in the GMT group appeared to endorse higher subjective ratings of cognitive and functional impairment on most measures. Here, individuals who agreed to participate in the study after initial recruitment and randomization (i.e., the GMT group) may have exhibited greater treatment-seeking behavior, potentially enhancing treatment gains. By contrast, only three of the waitlist participants sought placement in a GMT group following completion of their final assessment, pointing toward decreased treatment motivation.

On balance, participants in the treatment group showed improvements in planning problem solving, impulsivity/inhibitory control, sustained attention, and verbal memory that were not observed in a matched wait group. Critically, these treatment gains were accompanied by subjective reports of improved cognitive functioning on measures with items primarily pertaining to executive function and general memory. The observed translation of gains in neuropsychological performance to improvements in functioning is in keeping with the proposed generalizability of GMT, suggesting that cognitive improvements stemming from participation in the group are linked to improved performance on everyday tasks required for successful functioning (e.g., at home and at work). Participation in the GMT group was also associated with broadly positive responses at the exit interview, pointing again to its potential utility in an OCD sample. Future work, however, is urgently required to identify the mechanisms by which this protocol leads to the cognitive, functional, and subjective improvements observed in the present study. It is notable here that corresponding improvements were not observed in core symptoms of OCD, suggesting that improvements in disease severity cannot account for the neuropsychological, functional, and subjective gains observed here.

It is unlikely that cognitive remediation will be necessary for all individuals diagnosed with OCD, but given the promising results seen in this early investigation, it appears appropriate that it be offered to those who report subjectively impairment in daily cognitive functioning. Future approaches may also involve fewer, condensed sessions and might serve most effectively as a primer to, or in conjunction with current standard cognitive behavioral therapies. Given that Goal Management Training is already an established protocol and results of the exit interview from this pilot study showed that participants tended to enjoy the program content, the current program would likely provide the foundations for an adapted cognitive remediation protocol with a brief structure and more OCD-specific material.

There are several limitations to this study that may serve to inform future investigation. Despite lacking power, this small pilot sample served to adequately demonstrate the potential positive effects of cognitive remediation in this population while also pointing toward changes that may be necessary to the program structure and content. A small sample is inherent in a pilot setting, and an emphasis should be placed on effect size rather than significance of results, where an underpowered sample is to be expected. A crossover design to increase sample size was not adopted due to limited time and clinical resources, and the primary focus was instead on a preliminary pilot investigation of this protocol, but this option should be considered for future clinical trials. Furthermore, upon review of consistent participant feedback from the exit interview that many participants felt the protocol to be repetitive or lengthy, it was deemed unnecessary to continue to administer a protocol with the knowledge that it requires multiple revisions to be more suitable for this population.

One major limitation is the potential for type I error due to the large number of multiple comparisons (refer to Table 3 for a list of all measures included in the assessment), a problem often inherent with use of neuropsychological tasks involving multiple subscales—although it can be argued that many of these variables represent distinct processes at different levels of functioning. In addition, the majority of our sample had recently completed a course of cognitive behavioral therapy for OCD, and 74% were taking medication for OCD symptoms. The findings presented here, therefore, might not be generalizable to a wider or treatment-naïve population. However, we would expect that, if anything, a sample not treated previously would be more likely to show greater improvement in functioning over the course of treatment. Furthermore, although the retention rate of participants from pre- to post-treatment was high, there was significant attrition for the 3-month follow-up assessment (*n* = 19 to *n* = 6) and as such data on persistence of treatment effects is limited. The attrition was due in large part to lack of motivation for participants to attend a third assessment 3 months following the completion of their treatment. This will be of primary importance for future large-scale trials. The likely reason for this was that the majority of participants had already completed a course of CBT in addition to completing the cognitive remediation program, and had very little motivation to return for a final session 3 months following completion of these treatments. The simplest remedy to this would have been to offer a larger compensation for participation (e.g., $50 CAD rather than $20) particularly given the time commitment necessary for participants to attend an in-person appointment. Finally, due to resource and personnel limitations, full blinding was not always feasible. Where possible, the research team completed assessments by individuals blind to participant condition, but when this was not the case, there was potential for bias to have affected final results.

## Conclusions

The results of this pilot study, although tempered by the pilot sample size and other limitations mentioned above, are in line with our predictions surrounding the potential benefits of GMT in individuals with OCD and align closely with previous studies of GMT in various populations in that we observed focused, specific improvements in neuropsychological performance on variables of planning, problem-solving, impulsivity and attention, and general improvements in ratings of subjective cognition and daily functioning, particularly on domains related to cognition/understanding and instrumental daily functioning. The objective of this study was to assess the suitability of the GMT protocol in an OCD sample and to assess areas of improvement for this program’s implementation in this population and this objective was met. The Goal Management Training protocol in its current form is likely delivered over more sessions than necessary for most individuals with OCD, but will serve as an excellent base for the adaptation or development of a new protocol with material specifically tailored toward this population, which will be the focus of future trials. These encouraging findings can inform future research, which should investigate a more condensed cognitive remediation format with larger sample and rigorous study design. Future study should also consider the addition of an active control group in order to determine specific effects of the cognitive remediation program relative to other treatment options. Though likely not necessary for all individuals with OCD, the findings of this preliminary investigation suggest that the utility of cognitive remediation strategies should be investigated as they may confer significant gains for interested individuals particularly those who are experiencing difficulty with cognitive function.

## Data Availability

Transfer of data outside the housing institution is currently not supported by the institution’s ethics policy.

## Abbreviations

ADHD: Attention-deficit hyperactivity disorder
CBT: Cognitive behavioral therapy
CFQ: Cognitive Failures Questionnaire
CPT: Conners’ Continuous Performance task
CSTC: Cortico-striato-thalamo-cortical
CVLT-II: California Verbal Learning Test, second edition
DASS-21: Depression Anxiety and Stress Scales 21-item version
DEX: Dysexecutive Questionnaire
DSM-5: Diagnostic and Statistical Manual of Mental Disorders 5th Edition
GMT: Goal Management Training
IIRS: Illness Intrusiveness Rating Scale
MACCS: Memory and Cognitive Confidence Scale
OCD: Obsessive-compulsive disorder
RCFT: Rey-Osterrieth Complex Figure Task
SDS: Sheehan Disability Scale
SSRI: Selective serotonin reuptake inhibitor
TOL: Tower of London
WASI: Wechsler Adult Intelligence Scale
WHODAS: World Health Organization Disability Assessment Schedule 2.0
WLC: Waitlist control group
WTAR: Wechsler Test of Adult Reading
Y-BOCS: Yale-Brown Obsessive-Compulsive Scale
